# Access to Abundant Resources Mitigates the Effects of Nutritional Status on Life History Trade‐Offs: An Experimental Study on Burying Beetles

**DOI:** 10.1002/ece3.72210

**Published:** 2025-09-22

**Authors:** Wenxia Wang, Guojun Zhou, Kai Tian, Lunguang Yao, Jan Komdeur

**Affiliations:** ^1^ Henan Field Observation and Research Station of Headwork Wetland Ecosystem of the Central Route of South‐to‐North Water Diversion Project, College of Life Sciences Nanyang Normal University Nanyang China; ^2^ Behavioral and Physiological Ecology, Groningen Institute for Evolutionary Life Sciences (GELIFES), Faculty of Science and Engineering University of Groningen Groningen the Netherlands; ^3^ Nanyang Medical College Nanyang China; ^4^ Henan International Joint Laboratory of Soil Health and Water Security, College of Life Sciences Nanyang Normal University Nanyang China

**Keywords:** life history trade‐offs, *Nicrophorus vespilloides*, nutritional status, resource acquisition, sexual maturation

## Abstract

Individuals can adjust allocation strategies based on intrinsic and extrinsic factors. Variation in nutritional status during sexual maturation and resource acquisition during breeding may affect life history functions and trade‐offs. However, it is unclear whether individuals can compensate for poor nutritional status during sexual maturation when resource acquisition becomes favorable during breeding. To investigate this, we simultaneously manipulated male nutritional status during sexual maturation and resource acquisition during breeding in a burying beetle ( *Nicrophorus vespilloides* ). We monitored the separate and interactive effects of these two factors on life history functions and trade‐offs (somatic vs. reproductive investment, number vs. size of offspring). We found that poor nutritional status during sexual maturation negatively affects reproductive investment in adulthood, whereas access to abundant resources during breeding buffers against these effects. Poor‐fed males mitigated the initial differences in nutritional status compared to well‐fed males by feeding more from the carcass, and thus masking the long‐term effects of nutritional status on the reproductive performance. They gained more weight but provided less care than well‐fed males when breeding on a small carcass, whereas they gained more weight and provided a similar amount of care as well‐fed males when breeding on a large carcass. In addition, nutritional status during sexual maturation had no significant effects on subsequent reproductive performance (larvae number and average larval mass). However, there were significant trade‐offs between the number and size of offspring, suggesting that poor‐fed males consumed more from the carcass, which in turn reduced the amount of food resources for their offspring. The results obtained for males are independent of female partner's efforts and compensation effects. Our findings demonstrated that although the nutritional environment experienced during sexual maturation may influence allocation strategies in adulthood, individuals can compensate for a poor start if resource acquisition becomes more favorable during subsequent breeding.

## Graphical abstract

We manipulated male nutritional status during sexual maturation and resource acquisition during breeding in a burying beetle to assess their effects on life history functions and trade‐offs. We found that poor‐fed males mitigated the initial differences in nutritional status compared to well‐fed males by feeding more from the carcass, thereby masking the long‐term effects of nutritional status on the reproductive performance. The study reveals that although the nutritional status during sexual maturation may influence the allocation strategies in adulthood, individuals can compensate for a poor start if resource acquisition becomes favorable during subsequent breeding.

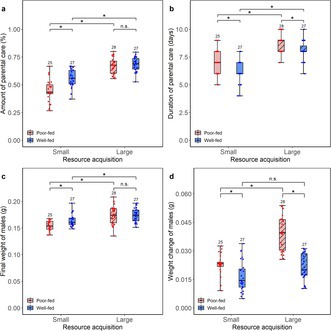

## 1 Introduction

Life history theory suggests that individuals should allocate resources between mutually exclusive functions (i.e., somatic and reproductive investment, current and future reproduction, the number and size of offspring), because any increased resource allocation towards one function will be associated with a reduced allocation towards the other (Van Noordwijk and De Jong 1986). Therefore, individuals are expected to evaluate multiple intrinsic (i.e., nutritional status: Keppner et al. 2023; Lambert and Smiseth 2024; age: Berger et al. 2015; Jehan et al. 2021; prior experience: Dubey et al. 2018; Antunes and Taborsky 2020) and extrinsic factors (i.e., resource acquisition: Richardson and Smiseth 2020; Winkler et al. 2025; predation or parasitism risk: Hoy et al. 2016; Albery et al. 2021) when making their allocation strategies. Several studies have tested how individuals adjust their life history functions and trade‐offs based on variation in nutritional status by manipulating the amounts of resources during early life or prior to breeding and then comparing the response of poor‐fed and well‐fed individuals (Wong and Kölliker 2012; Hayward et al. 2013; Holden et al. 2019; Keppner et al. 2023). It has been suggested that poor‐fed individuals typically exhibit impaired somatic maintenance (i.e., reduced growth rate, depressed immunity) and preferentially allocate resources to replenish their energy reserves by compensatory feeding, which leads to negative effects on their reproductive performance (Norris and Evans 2000; Auer et al. 2010; Holden et al. 2019). Therefore, poor‐fed individuals generally perform worse in reproduction (i.e., reduced mating success and fecundity, lower reproductive effort and offspring performance), whereas well‐fed individuals usually show increased reproductive investment (Wong and Kölliker 2012; Hayward et al. 2013; Holden et al. 2019; Keppner et al. 2023). Previous studies have also shown that nutritional status is commonly considered as a proxy of fitness and can mediate life history trade‐offs (Snell‐Rood et al. 2015; Swanson et al. 2016). Specifically, poor‐fed individuals may exhibit more pronounced life history trade‐offs, whereas well‐fed individuals can afford to allocate resources to multiple traits, thereby mitigating the underlying trade‐offs (Reznick et al. 2000; Snell‐Rood et al. 2015; Swanson et al. 2016). Furthermore, variation in nutritional status during sexual maturation may be more sensitive than other stages, as it has long‐term effects on adult morphology or reproductive strategy (Barrett, Moore, and Moore 2009; Barrett, Hunt, et al. 2009; Richardson and Smiseth 2019; Hopwood et al. 2013). Previous studies have suggested that poor nutritional status during sexual maturation can have lasting negative effects on the physiology, fertility, and reproductive success of adults (Barrett, Moore, and Moore 2009; Barrett, Hunt, et al. 2009; Holden et al. 2019; Wilner et al. 2020). As such, individuals may therefore perform different allocation strategies and express alternative life history trade‐offs.

In addition, variation in resource acquisition during breeding should have an important impact on life history functions and trade‐offs given that it determines how many resources can be allocated to different traits. It has been suggested that large amounts of resources may mitigate the life history trade‐offs by masking the negative relationships between different functions, whereas limited resources may intensify such trade‐offs (Van Noordwijk and De Jong 1986; King et al. 2011; Winkler et al. 2025). Although we have a good understanding of the influences of nutritional status and resource acquisition on life history functions and trade‐offs separately, it remains unknown whether individuals can compensate for a poor start (i.e., poor nutritional status during sexual maturation) when resource acquisition becomes more favorable during subsequent breeding. Thus, there is now a need for more studies to examine the potential interactive effects of nutritional status during sexual maturation and resource acquisition during breeding on life history functions and trade‐offs. In this study, we use the burying beetle ( *Nicrophorus vespilloides* ) in which we simultaneously manipulated nutritional status during sexual maturation and subsequent resource acquisition during breeding to assess their separate and interactive effects on somatic and reproductive investment and trade‐offs between the number and size of offspring.

Burying beetles breed on small vertebrate carcasses which are unpredictable in the wild and serve as the sole food resources for both parents and their offspring during breeding (Scott 1998). Parents provide flexible parental care for their offspring, including (i) the carcass preparation, in which parents prepare the carcass by removing hair, rolling the carcass into a ball underground, and applying anti‐microbial secretions on its surface to delay decomposition (Eggert et al. 1998; Scott 1998; Rozen et al. 2008; Trumbo 2017); (ii) the larvae provisioning, in which parents feed their larvae with pre‐digested carcass (Eggert et al. 1998; Scott 1998; Smiseth et al. 2003; Potticary et al. 2024). After hatching, the larvae crawl towards the carcass and obtain food by self‐feeding or begging (Smiseth and Moore 2002; Smiseth et al. 2003; Capodeanu‐Nägler et al. 2018). Subadult burying beetles must feed for several days post‐eclosion (approximately 10 days) to attain sexual maturation, and they cannot increase body size during this period (Wilson and Knollenberg 1984). Therefore, this creates the unique opportunity to manipulate nutritional status during sexual maturation independent of adult body size. Prior works have reported that burying beetle adult males that were starved during sexual maturation spend more time signaling for females (Richardson and Smiseth 2019), suggesting that nutritional status during sexual maturation may have an important effect on their allocation strategies. In addition, burying beetle males improve their reproductive investment as carcass size increases during breeding (Luzar et al. 2017; Wang et al. 2022). As such, it is expected that the long‐term effects of nutritional status during sexual maturation on their allocation strategies in adulthood might be affected by subsequent resource acquisition during breeding. However, few studies have simultaneously manipulated these two factors and examined their interactive effects on life history functions and trade‐offs. By monitoring the interactive effects of nutritional status and resource acquisition, it can be revealed whether individuals can compensate for a poor start during subsequent breeding.

In this study, we aimed to examine how burying beetle males adjust life history functions and trade‐offs based on both their nutritional status during sexual maturation and resource acquisition during breeding. To this end, we provided males with different kinds of food supply during sexual maturation (nutritional status: poor (mealworm) vs. well (mouse carcass)) and different‐sized mouse carcasses (resource acquisition: small vs. large) during breeding. We then monitored the separate and interactive effects of nutritional status and resource acquisition on their somatic and reproductive investment (final weight, weight change, the amount and duration of parental care), and offspring performance (larvae number, average larval mass, trade‐offs between the number and size of larvae). We expect an interactive effect between nutritional status and resource acquisition on life history functions or trade‐offs. Poor‐fed males may compensate for a bad start and perform as well as well‐fed males in reproduction when resource acquisition becomes more favorable during breeding. When resources are limited, poor‐fed males would perform worse in reproduction and invest more in somatic maintenance than well‐fed males (Keppner et al. 2023). Specifically, we expect that poor‐fed males will spend more time preparing the carcasses, consume more from the carcasses, thus produce fewer and smaller offspring, and provide less care than well‐fed males. When resources are abundant, poor‐fed males would have more chances to invest in both somatic maintenance and reproduction (Smiseth et al. 2014; Keppner et al. 2018). Therefore, they are expected to replenish their energy reserves by feeding more from the carcass, produce the same broods, and provide the same amount of care as well‐fed males. We also predicted that males would gain more weight, produce more and heavier offspring, and provide more care as resources increase (Smiseth et al. 2014). Finally, we expected that males, irrespective of nutritional status during sexual maturation, may have a chance to avoid trade‐offs between the number and size of offspring when resources during breeding are abundant (Smiseth et al. 2014).

## 2 Materials and Methods

### 2.1 Beetle Colony

All burying beetles used for this study were laboratory‐reared second‐/third‐generation offspring of adults collected in August 2023 from the Baotianman National Nature Reserve, Neixiang, China. All adults and larvae were reared in the College of Life Science at Nanyang Normal University and were kept in plastic boxes (length: 20 cm; width: 15 cm; height: 10 cm) filled with moist soil under a 16:8 h light:dark cycle at 24°C ± 2°C. All non‐breeding beetles were fed with 2–3 freshly decapitated mealworms ( *Tenebrio molitor* ) twice per week, with 2–3 days' intervals between feedings. We maintained a large stock population of 300–400 individuals per generation (approximately 25 families) and recruited only three individuals from each family to establish the next generation. We also randomly paired burying beetles that were neither cousins nor siblings to avoid inbreeding. All experiments were conducted from October to December 2023.

### 2.2 Manipulation of Nutritional Status and Resource Acquisition

We manipulated the nutritional status of males during sexual maturation (mealworm‐fed vs. mouse carcass‐fed) and resource acquisition during breeding (mouse carcass amount of 8.34 ± 0.58 g vs. 15.41 ± 0.72 g). To start the experiment, we placed the subadult males (*N* = 120) individually in plastic boxes filled with approximately 1.5 cm soil for 2 weeks post‐eclosion. During this period, half of the males (*N* = 60) were fed with freshly decapitated mealworms ( *T. molitor* ) at the rate of 2–3 mealworms per beetle, twice weekly with 2–3 days intervals between feedings. The other half of the males (*N* = 60) were fed once with a freshly thawed baby mouse carcass ( *Mus musculus* ), 3–5 g per beetle. The purpose of these treatments was to generate males that differed in their nutritional status during sexual maturation. We recorded the body weight (accuracy: 0.0001 g) of males before and after the nutritional treatments to confirm whether the manipulations altered their nutritional status. We chose these nutritional status treatments because mealworms meet their developmental needs, and carcasses are superior resources for burying beetles (Scott 1998). We chose 2 weeks as the treatment duration to avoid behavioral variation due to any possible delay in sexual maturation because *N. vespilloides* can breed after 14 days post‐eclosion when maintained on the same feeding protocol as the mealworm‐fed beetles based on previous studies (Wang et al. 2025). We then randomly paired each male with an unrelated virgin female aged 2 weeks post‐eclosion and provided them with a mouse carcass (small vs. large carcass, 8.34 ± 0.58 g vs. 15.41 ± 0.72 g) for breeding. We chose these treatment levels based on the fact that an 8.34 ± 0.58 g carcass is a relatively small treatment level to ensure burying beetles start their breeding, and a 15.41 ± 0.72 g carcass would be completely consumed at the time of larval dispersal (Richardson and Smiseth 2020; Wang et al. 2022).

### 2.3 Observation of Parental Investment and Offspring Performance

Once the carcass was added to the boxes, we checked the parental care behavior of parents three times daily (07:00–08:00 am, 13:00–14:00 pm, 19:00–20:00 pm, 5‐h intervals) by visual inspection. In burying beetles, the presence on (carcass maintenance) or inside (larvae provisioning) the carcass is a strong indicator of parental care (Walling et al. 2008; Head et al. 2014). We therefore recorded whether parents were present on or inside the carcass, or whether they were invisible in the soil during each check by carefully removing the surface soil of the carcass until larval dispersal. We then estimated the amount of parental care as the proportion of times that parents were present on or inside the carcass of the total observation times. We calculated the duration of parental care as the number of days parents were present on or in the carcasses until their desertion (absent from the carcass for three consecutive observations) or until larvae dispersal (Benowitz et al. 2013; Head et al. 2014). After larvae dispersal, we counted the larvae number and weighed the total brood mass of each brood (accuracy: 0.01 g), then calculated the average larval mass as the total brood mass divided by the larvae number. We measured the final body weight of parents at the time of terminating care or at the time of larval dispersal to calculate their weight change during the entire breeding, because this is an indicator of the somatic investment in burying beetles (Trumbo and Xhihani 2015; Ratz et al. 2021). We also measured the pronotum width (accuracy: 0.01 mm) of parents, because large parents are predicted to provide more care and produce larger offspring (Steiger 2013; Pilakouta et al. 2015).

### 2.4 Statistical Analyses

All analyses were performed using R version 4.0.3 (R Core Team 2020) loaded with packages *car*, *lme4*, *emmeans*, *DHARMa*. We used linear models (LMs) for traits that had a normal structure (initial weight, final weight, weight change, average larval mass at dispersal), generalized (mixed) linear models for traits that had a Poisson error distribution (GLMs: larvae number, GLMMs: the duration of parental care) and Binomial error distribution (GLMMs: the amount of parental care). All full models included the fixed factors of male nutritional status (poor‐fed, well‐fed), resource acquisition (small, large), and the interaction between them. Group identity was included as a random factor in GLMs and GLMMs. In the models for parental care, we included the partner's effort and larvae number at dispersal as covariates because males may adjust their reproductive investment based on their partner's investment and brood size (Johnstone and Hinde 2006; Pilakouta et al. 2015; Wang et al. 2021; Sahm et al. 2023). In the models for weight change, we included the partner's weight change as a covariate because males may adjust their carcass consumption based on their partner's consumption (Pilakouta et al. 2016). These covariates were included in the final models when they significantly improved the model fit. We did this by comparing models in which these factors were included or excluded as additional effects. We compared the significance of the fixed and interactive effects across models that included or excluded these covariates to verify that the results were independent of them. We used the *Anova* function to obtain Chi‐square and *p* values, and *post hoc* pairwise comparisons to test differences whenever the interaction had significant effects on the variables. We applied Bonferroni corrections to minimize type I errors for multiple tests (Rice 1989). We estimated the over/under‐dispersion of the models by using the *testDispersion* function and verified the good fit of the models by using the *simulateResiduals* function of *DHARMa*. We excluded trials from our analyses in which males died during the nutritional manipulations or before larvae dispersal, or females failed to lay eggs, which yielded the following sample size of our experiment: poor‐fed males (8.34 ± 0.58 g, *N* = 25; 15.41 ± 0.72 g, *N* = 28), well‐fed males (8.34 ± 0.58 g, *N* = 27; 15.41 ± 0.72 g, *N* = 27) during sexual maturation.

## 3 Results

### 3.1 Effects of Nutritional Treatments on the Body Weight of Burying Beetle Males

The initial body weight and pronotum width of females and subadult males did not differ among different treatments and were therefore excluded from subsequent analyses (Tables 1 and S1). The body weight of poor‐fed males was significantly lower than that of well‐fed males after the nutritional manipulations (Tables 1 and S1), confirming that they were in different nutritional statuses. The mean body weight of poor‐fed males was 11.44% lower than that of well‐fed males at the end of nutritional manipulations. Poor‐fed and well‐fed males gained 5.29% and 22.57% of their body weight at eclosion after nutritional treatments, respectively, thereby mitigating the effects of starvation on the time males spent on the carcass.

**TABLE 1:** Summary of two‐way ANOVA for pronotum width and initial weight of burying beetle parents (the body weight at the start of breeding) among different nutritional statuses (poor‐fed vs. well‐fed) and resource acquisitions (small vs. large carcass).

Explanatory variable	Initial subadult male weight		Initial male weight		Initial female weight		Male pronotum width		Female pronotum width	
	*F*	*p*	*F*	*p*	*F*	*p*	*F*	*p*	*F*	*p*
Nutritional status	0.505	0.479	57.361	**< 0.001**	0.426	0.516	1.092	0.299	1.142	0.288
Resource acquisition	0.682	0.412	2.396	0.125	0.282	0.596	0.014	0.907	0.249	0.619
Interaction	0.077	0.782	0.136	0.714	0.192	0.662	0.070	0.792	0.463	0.498

*Note:* Poor‐fed versus well‐fed, mealworm‐fed versus mouse carcass‐fed; Small versus large carcass, 8.34 ± 0.58 g versus 15.41 ± 0.72 g. Significant *p*‐values are indicated in bold. The sample size, mean value, and standard deviation are provided in Table S1.

### 3.2 Effects of Nutritional Status During Sexual Maturation, Resource Acquisition During Breeding, and Their Interaction on the Parental Care of Burying Beetle Males

**FIGURE 1:**
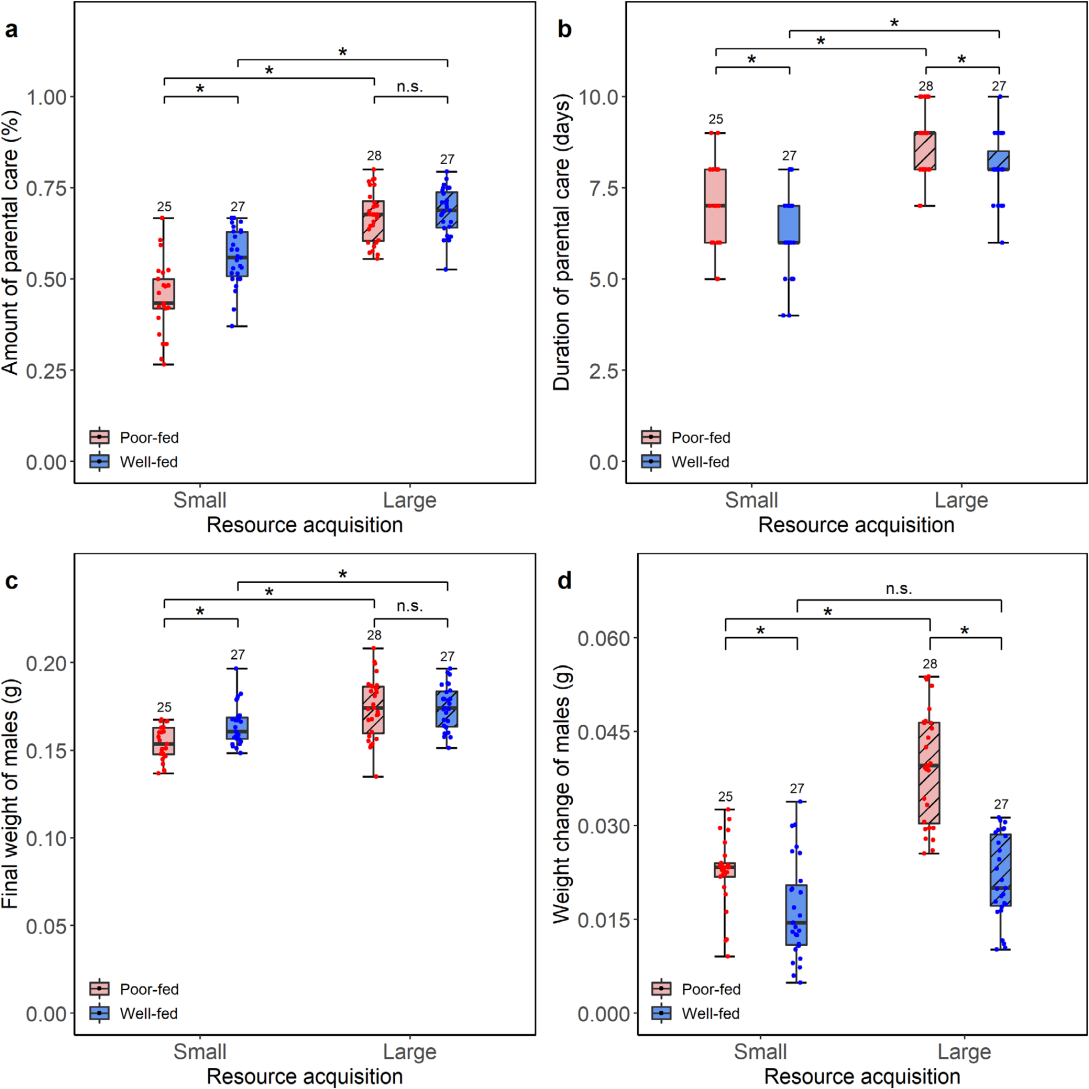
Effects of nutritional status during sexual maturation (poor‐fed vs. well‐fed), resource acquisition during breeding (small vs. large carcass), and their interaction on (a, b) the amount and duration of parental care, and (c, d) the final weight and weight change in male burying beetles. Poor‐fed versus well‐fed, mealworm‐fed versus mouse carcass‐fed. Small versus large carcass, 8.34 ± 0.58 g versus 15.41 ± 0.72 g. Numbers above error bars are sample sizes. Boxplots show median, interquartile range, and minimum/maximum range. Asterisks, significant (*p* < 0.05); n.s., not significant.

In the models for parental care, we included the partner's effort as covariates in the final models because males adjusted the amount (χ^2^ = 7.428, *p* = 0.006) and duration (χ^2^ = 37.937, *p* < 0.001) of care based on their partner's effort. The results were independent of the partner's effort, as the significance of the fixed and interactive effects remained unchanged regardless of whether these covariates were included or excluded (Table 2a,b). The larvae number had no significant effects on the amount (χ^2^ = 1.049, *p* = 0.306) and duration (χ^2^ = 0.008, *p* = 0.929) of care in burying beetle males and was therefore excluded from the final models. There was a significant interactive effect between nutritional status during sexual maturation and resource acquisition on the amount of care during breeding (Table 2, Figure 1a). Specifically, poor‐fed males provided less care than well‐fed males when breeding on a small carcass (poor‐fed vs. well‐fed: Estimate = −0.410, *p* = 0.006), whereas they provided the same amount of care as well‐fed males when breeding on a large carcass (poor‐fed vs. well‐fed: Estimate = −0.040, *p* = 0.999). In addition, we found that resource acquisition had significant effects on the amount and duration of care during breeding (Table 2, Figure 1b). Males provided more care and deserted later when breeding on large versus small carcasses. Poor‐fed males stayed for longer on the carcass than well‐fed males (Table S2). Finally, after excluding the fixed factors of male nutritional status from the models, we found that the body weight of males by the end of sexual maturation had a significant positive effect on the amount of care (χ^2^ = 7.061, *p* = 0.008) but a negative effect on the duration of care (χ^2^ = 12.035, *p* = 0.001) in burying beetle males. Males that attained higher body weight by the end of sexual maturation provided more parental care but deserted the brood earlier compared to males with lower body weight (Tables S1 and S2).

**TABLE 2:** Effects of nutritional status during sexual maturation (poor‐fed vs. well‐fed), resource acquisition during breeding (small vs. large carcass) of male burying beetles, and their interaction on the amount and duration of parental care (a, included the partner's effort as covariates; b, excluded the partner's effort as covariates).

Explanatory variable	(a)				(b)			
	Amount of parental care		Duration of parental care		Amount of parental care		Duration of parental care	
	*χ* ^2^	*p*	*χ* ^2^	*p*	*χ* ^2^	*p*	*χ* ^2^	*p*
Nutritional status	8.681	**0.003**	45.615	**< 0.001**	11.785	**0.001**	18.377	**< 0.001**
Resource acquisition	94.738	**< 0.001**	41.459	**< 0.001**	97.133	**< 0.001**	96.529	**< 0.001**
Interaction	6.517	**0.011**	0.523	0.469	7.154	**0.008**	0.602	0.438

*Note:* Poor‐fed versus well‐fed, mealworm‐fed versus mouse carcass‐fed; Small versus large carcass, 8.34 ± 0.58 g versus 15.41 ± 0.72 g. Degree of freedoms are all equal to 1; Significant *p*‐values are indicated in bold. The sample size, mean value, and standard deviation are provided in Table S2.

### 3.3 Effects of Nutritional Status During Sexual Maturation, Resource Acquisition During Breeding, and Their Interaction on the Final Weight and Weight Change of Burying Beetle Males

The partner's final weight or weight change had no significant effects on the final weight (*F* = 0.025, *p* = 0.874) or weight change (*F* = 1.296, *p* = 0.258) of males, and was therefore excluded from the final models. There was a significant interactive effect between nutritional status during sexual maturation and resource acquisition on the final weight (weight at the end of breeding) and weight change of males during breeding (Table 3, Figure 1c,d). The final weight of poor‐fed males was lighter than well‐fed males when breeding on a small carcass (poor‐fed vs. well‐fed: Estimate = −0.011, *p* = 0.031), whereas there was no difference in the final weight between poor‐fed and well‐fed males when breeding on a large carcass (poor‐fed vs. well‐fed: Estimate = 0.000, *p* = 0.999). The poor‐fed and well‐fed males differed significantly in weight change when carcass mass increased from small to large. That is, poor‐fed males gained more weight when breeding on large versus small carcass (small vs. large: Estimate = 0.017, *p* < 0.001), whereas well‐fed males gained the same weight regardless of the amount of resource during breeding (small vs. large: Estimate = 0.005, *p* = 0.083). In addition, we found that nutritional status had a significant effect on the weight change of males during breeding. Poor‐fed males gained more weight than well‐fed males during breeding. Finally, we found that the amount (*F* = 2.291, *p* = 0.133) and duration (*F* = 0.672, *p* = 0.414) of parental care had no significant effects on the weight change of males.

**TABLE 3:** Effects of nutritional status during sexual maturation (poor‐fed vs. well‐fed), resource acquisition during breeding (small vs. large carcass) of male burying beetles, and their interaction on final weight (the body weight of males after breeding), weight change (the weight change of males during breeding), larvae number, and average larval mass at dispersal.

Explanatory variable	Final weight		Weight change		Larvae number		Average larval mass	
	*F*	*p*	*F*	*p*	*χ* ^2^	*p*	*χ* ^2^	*p*
Nutritional status	4.025	**0.047**	70.115	**< 0.001**	0.416	0.518	0.123	0.726
Resource acquisition	35.006	**< 0.001**	58.018	**< 0.001**	56.145	**< 0.001**	5.979	**0.016**
Interaction	4.119	**0.045**	17.140	**< 0.001**	0.176	0.675	0.037	0.847

*Note:* Poor‐fed versus well‐fed, mealworm‐fed versus mouse carcass‐fed; Small versus large carcass, 8.34 ± 0.58 g versus 15.41 ± 0.72 g. Degree of freedoms are all equal to 1; Significant *p*‐values are indicated in bold. The sample size, mean value, and standard deviation are provided in Table S2.

### 3.4 Effects of Nutritional Status During Sexual Maturation, Resource Acquisition During Breeding, and Their Interaction on the Offspring Performance

**FIGURE 2:**
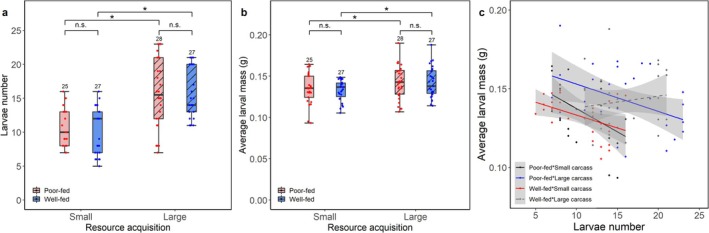
Effects of nutritional status during sexual maturation (poor‐fed vs. well‐fed), resource acquisition during breeding (small vs. large carcass) of male burying beetles, and their interaction on (a) the larvae number, (b) average larval mass, and (c) the relationship between larvae number and average larval mass. Poor‐fed versus well‐fed, mealworm‐fed versus mouse carcass‐fed. Small versus large carcass, 8.34 ± 0.58 g versus 15.41 ± 0.72 g. Numbers above error bars are sample sizes. Boxplots show median, interquartile range, and minimum/maximum range. Asterisks, significant (*p* < 0.05); n.s., not significant. The solid lines represent the significant negative relationships (poor‐fed * small carcass, *p =* 0.017; poor‐fed * large carcass, *p* = 0.024; well‐fed * small carcass, *p* = 0.019), and dashed regression lines show non‐significant relationships (well‐fed * large carcass, *p =* 0.46).

We found that resource acquisition during breeding, but not nutritional status during sexual maturation, had significant effects on larval number and average larval mass at dispersal (Table 3, Figure 2a,b). Parents produced more and heavier larvae when breeding on large versus small carcasses, whereas there was no significant difference in larval number and average larval mass among different nutritional status treatments (Table S2). Finally, only when well‐fed males bred on large carcasses, was there was no significant negative relationship between larval number and average larval mass at dispersal (Table 3, Figure 2c).

## 4 Discussion

In this study, we investigated the separated and interactive effects of nutritional status during sexual maturation (a key developmental window) and resource acquisition during breeding on life history functions and trade‐offs in burying beetle males. The results below are independent of partner's effort, as the significance of both fixed and interactive effects remained consistent regardless of including or excluding these covariates. We found that poor nutritional status resulted in preferential allocation to somatic maintenance but not reproduction. However, access to abundant resources during breeding buffers against the effects of nutritional status on life history functions and trade‐offs. Poor‐fed males gained more weight and provided less care than well‐fed males when breeding on a small carcass, whereas they replenished energy reserves and provided the same amount of care as well‐fed males when breeding on a large carcass. Meanwhile, abundant resources allowed males to invest more into somatic maintenance without incurring costs to their larvae. Below we provided a detailed discussion of our results.

### 4.1 Effects of Nutritional Status During Sexual Maturation, Resource Acquisition During Breeding, and Their Interaction on the Parental Care of Burying Beetle Males

As predicted, we found that resource acquisition influenced the effects of nutritional status on life history functions. When resources were limited, poor‐fed males provided less care and stayed for longer on the carcass than well‐fed males. It may be because poor‐fed males spend more time preparing the carcass. This result is in line with previous nutritional manipulation studies that burying beetles that had been starved during sexual maturation performed worse on adult behavior (i.e., competitive ability: Hopwood et al. 2013; sexual attractiveness: Richardson and Smiseth 2019). Furthermore, we suggest that although poor‐fed males are able to recover energy by compensatory feeding when breeding on a small carcass, they do not fully catch up to well‐fed males in the amount of parental care. When resources were abundant, poor‐fed males provided a similar amount of care as well‐fed males. Considering that burying beetle parents gain benefits by feeding from the carcass during breeding (Creighton et al. 2009; Lambert and Smiseth 2022), a potential explanation is that poor‐fed males have more chance to recover energy when breeding on a large carcass. That is, they may buffer against the initial differences in nutritional status with well‐fed males by feeding from the carcass. The assumption was also supported by the fact that poor‐fed males stayed for longer on the carcass and gained more weight than well‐fed males. However, the duration of parental care had no significant effect on the weight change of males, suggesting that the extended time spent on the carcass is not the sole strategy to gain weight for poor‐fed males. Moreover, we suggest that nutritional status during sexual maturation influenced the reproductive investment of burying beetle males by affecting their body weight, given that their body weight at the end of sexual maturation had significant effects on the amount and duration of care. In this case, the long‐term effects of nutritional status during sexual maturation may diminish with time as males gain benefits from the carcass. Another potential explanation may be that the effects of nutritional status were reduced in the laboratory, because males did not have to search and compete for carcasses and partners. It is costly to compete for and protect the carcass in the wild (Scott 1998; Eggert and Sakaluk 2000; Hopwood et al. 2015; Luzar et al. 2017; Potticary et al. 2024). We also found that both poor‐fed and well‐fed males provided more care and stayed for longer when breeding on large versus small carcasses, suggesting that large carcasses required more time to prepare and preserve (Scott 1998; Trumbo 2017; Luzar et al. 2017). Alternatively, it could reflect that the time males spent on the small carcass was restricted by their partners. This is because burying beetle females have been observed attempting to drive males away from the carcass to conserve more of the food resources for themselves and their developing larvae (Keppner et al. 2018). Additionally, we found that males adjust the amount and duration of parental care based on their partner's effort, which is consistent with previous studies on burying beetles (Wang et al. 2021).

### 4.2 Effects of Nutritional Status During Sexual Maturation, Resource Acquisition During Breeding, and Their Interaction on the Final Weight and Weight Change of Burying Beetle Males

The final weight and weight change of males were independent of their partner's final weight and weight change, as the covariates showed non‐significant effects and were thus excluded from the final models. We found that poor‐fed males gained more weight as carcass mass increased during breeding, whereas well‐fed males gained the same weight regardless of carcass mass. It may be because poor‐fed males require more resources to recover energy, and a large carcass allows them to consume more and gain more weight. In contrast, well‐fed males may not require as much energy as poor‐fed males; thus, a small carcass can meet the requirements of resource consumption very well as a large carcass. We also found that poor‐fed males gained more weight than well‐fed males during breeding, indicating that poor‐fed males used the breeding attempt as an opportunity to recover energy. This result is consistent with previous studies that poor‐fed burying beetles feed more from the carcass during breeding than well‐fed burying beetles (Keppner et al. 2018, 2023). Considering the result that poor‐fed males provided less care than well‐fed males when breeding on a small carcass, we suggest that poor‐fed males showed compensatory feeding by allocating more resources into somatic maintenance at the expense of reduced reproductive investment. However, the benefits poor‐fed males gained by feeding from the large carcass can buffer against the initial differences in their nutritional status with well‐fed males without resulting in reduced reproductive investment when breeding on a large carcass.

### 4.3 Effects of Nutritional Status During Sexual Maturation, Resource Acquisition During Breeding, and Their Interaction on the Offspring Performance

Our results are consistent with previous studies that parents produce more and heavier larvae when breeding on a larger carcass (Smiseth et al. 2014; Ratz et al. 2021; Wang et al. 2022), suggesting that larger carcasses allow males to consume more resources without incurring costs to their larvae. We found no evidence that variation in male nutritional status had any effects on larvae number and average larval mass. However, there were significant trade‐offs between the number and size of offspring, suggesting that poorly fed males consumed more from the carcass, which in turn reduced the amount of food resources for their offspring (Keppner et al. 2018). Only when well‐fed males bred on large carcasses were there were no significant trade‐offs between larvae number and average larval mass at dispersal, indicating that large carcasses provided enough energy to allocate to both offspring number and size and thus mitigated such trade‐offs.

## 5 Conclusions and Recommendations for Future Research

In summary, resource acquisition during breeding influenced the effects of nutritional status during sexual maturation on life history functions and trade‐offs. When resources were limited, poor‐fed males performed worse in reproduction and invested more in somatic maintenance than well‐fed males. When resources were abundant, poor‐fed males compensated for a bad start and provided the same amount of care as well‐fed males. That is, poor‐fed males used the breeding attempt as an opportunity to recover energy; the benefits they gained by feeding from the carcass can buffer against the initial differences in their nutritional status with well‐fed males when resource acquisition becomes more favorable during breeding. In this study, we utilized the biparental care system of burying beetles but only focused on males to investigate the separate and interactive effects of nutritional status and resource acquisition on life history trade‐offs. This is because this system reflects the natural conditions where uniparental male care is extremely rare (approximately 3% in *N. vespilloides* , Eggert 1992), thereby making it more suitable for investigating the responses of males. Conversely, the prevalence of uniparental female care makes it a more appropriate model for studies focused on females (Wang et al. 2025). Our findings have important implications for understanding the interactive effects of nutritional status during a key developmental window and resource acquisition during breeding on life history functions and trade‐offs in burying beetles. However, we did not investigate the effects of nutritional status during larval development (which determines the body size of adults), which may also have important effects on their allocation strategies. We thus advocate that future studies should also include the effects of nutritional status during the early larval development period.

## Author Contributions

**Wenxia Wang:** conceptualization (equal), formal analysis (supporting), funding acquisition (lead), visualization (supporting), writing – original draft (lead). **Guojun Zhou:** conceptualization (equal), data curation (lead), formal analysis (lead), writing – original draft (supporting). **Kai Tian:** data curation (supporting), visualization (lead), writing – review and editing (supporting). **Lunguang Yao:** conceptualization (equal), supervision (lead), writing – original draft (supporting), writing – review and editing (equal). **Jan Komdeur:** formal analysis (supporting), writing – review and editing (lead).

## Conflicts of Interest

The authors declare no conflicts of interest.

## Supporting information


**Appendix S1:** ece372210‐sup‐0001‐AppendixS1.xlsx.

**Appendix S2:** ece372210‐sup‐0002‐AppendixS2.pdf.

**Appendix S3:** ece372210‐sup‐0003‐AppendixS3.pdf.

**Table S1:** Descriptive statistics for initial weight and pronotum width of burying beetle parents.
**Table S2:** Descriptive statistics for the amount and duration of parental care, final weight and weight change of burying beetle males, larvae number, and average larval mass at dispersal.

## Data Availability

Data and R code in this study are available as Appendices [Link], [Link].
