# Supplementary material for: Access to Abundant Resources Mitigates the Effects of Nutritional Status on Life History Trade‐Offs: An Experimental Study on Burying Beetles
Source: Ecol Evol. 2025 Sep 22;15(9):e72210. doi: 10.1002/ece3.72210 (PMC12453611; doi:10.1002/ece3.72210)
Supplement: Supplementary file 2 — Appendix S2: ece372210‐sup‐0002‐AppendixS2.pdf. [file ECE3-15-e72210-s001.pdf]

Access to abundant resources mitigates the effects of nutritional status on life history trade-offs: an experimental study on burying beetles.

Wenxia Wang, Guojun Zhou, Kai Tian, Lunguang Yao, Jan Komdeur

```
## Packages
library(car); library(lme4); library(emmeans)
## Data
a <- read_excel("Data.xlsx", col_types = c("text","text","numeric","numeric","numeric",
                              "numeric","numeric","numeric","numeric","numeric","numeric",
                              "numeric","numeric","numeric","numeric","numeric","numeric",
                              "numeric","numeric","numeric","numeric","numeric","numeric"))
```

Effects of nutritional status, resource acquisition, and their interaction on the amount and duration of parental care.

```
##the amount of parental care
M1_care <- glmer(cbind(male_care_times, male_observation_times - male_care_times) ~
                            resource_acquisition*nutritional_status + (1|id),
                    family = binomial(link = "logit"), data = a)
M2_care <- glmer(cbind(male_care_times, male_observation_times - male_care_times) ~
                            female_care_rate +
                            resource_acquisition*nutritional_status + (1|id),
                    family = binomial(link = "logit"), data = a)#covariate:the amount of female care
M3_care <- glmer(cbind(male_care_times, male_observation_times - male_care_times) ~
                          larvae_number +
                          resource_acquisition*nutritional_status + (1|id),
                 family = binomial(link = "logit"), data = a)#covariate:larvae number

anova(M1_care, M2_care)#the amount of female care was included in the final model
anova(M1_care, M3_care)#the larvae number was excluded in the final model
Anova(M2_care)
#interaction: resource_acquisition:nutritional_status
summary(contrast(emmeans(M2_care, ~ resource_acquisition*nutritional_status),method = "pairwise"),
        adjust = "Bonferroni")

##the duration of parental care
CareD1 <- (a$male_day)^2
CareD2 <- (a$female_day)^2
M1_careday <- glmer(CareD1 ~ resource_acquisition*nutritional_status + (1|id),
                         family = poisson(link = "log"), data = a)
M2_careday <- glmer(CareD1 ~ CareD2 +
                      resource_acquisition*nutritional_status + (1|id),
                    family = poisson(link = "log"), data = a)#covariate:the duration of female care
```

```
M3_careday <- glmer(CareD1 ~ larvae_number +
                      resource_acquisition*nutritional_status + (1|id),
                    family = poisson(link = "log"), data = a)#covariate:larvae number
anova(M1_careday, M2_careday)#the amount of female care was included in the final model
anova(M1_careday, M3_careday)#the larvae number was excluded in the final model
Anova(M2_careday)
```

Effects of males body weight by the end of sexual maturation on the amount and duration of parental care.

```
#the amount of care
M0_care <- glmer(cbind(male_care_times, male_observation_times - male_care_times) ~
                   resource_acquisition + initial_weight_male + (1|id),
                 family = binomial(link = "logit"), data = a)
Anova(M0_care)
summary(M0_care)#the body weight of males had a significant positive effect
                #on the amount of care (Est > 0)

#the duration of care
M0_careday <- glmer(CareD1 ~ resource_acquisition + initial_weight_male + (1|id),
                    family = poisson(link = "log"), data = a)
Anova(M0_careday)
summary(M0_careday)#the body weight of males had a significant negative effect
                   #on the duration of care (Est < 0)
```

Effects of nutritional status, resource acquisition, and their interaction on the final weight and weight change of males.

```
##the final weight of males
M1_FW <- lm(final_weight_male ~ resource_acquisition*nutritional_status, data = a)
M2_FW <- lm(final_weight_male ~ resource_acquisition*nutritional_status +
            final_weight_female, data = a)#covariate: the final body weight of females
anova(M1_FW, M2_FW)#the final body weight of females was excluded in the final model
Anova(M1_FW)
#interaction: resource_acquisition:nutritional_status
summary(contrast(emmeans(M1_FW, ~ resource_acquisition*nutritional_status),"pairwise"),
        adjust = "Bonferroni")

##the weight change of males
M1_WC <- lm(weight_change_male ~ resource_acquisition*nutritional_status, data = a)
M2_WC <- lm(weight_change_male ~ resource_acquisition*nutritional_status +
            weight_change_female, data = a)#covariate: the weight change of females
anova(M1_WC, M2_WC)#the weight change of females was excluded in the final model
Anova(M1_WC)
#interaction: resource_acquisition:nutritional_status
summary(contrast(emmeans(M1_WC, ~ resource_acquisition*nutritional_status),"pairwise"),
        adjust = "Bonferroni")
```

Effects of the amount and duration of parental care on the weight change of males.

```
M3_WC <- lm(weight_change_male ~ resource_acquisition*nutritional_status + male_day, data = a)
M4_WC <- lm(weight_change_male ~ resource_acquisition*nutritional_status + male_care_rate, data = a)
anova(M1_WC, M3_WC)
anova(M1_WC, M4_WC)
```

Effects of nutritional status, resource acquisition, and their interaction on the offspring performances.

```
LarvaeNumber1 <- glm(larvae_number ~ resource_acquisition*nutritional_status,
                    family = poisson(link = "log"), data = a)
Anova(LarvaeNumber1)
AverageMass1 <- lm(average_larvae_mass ~ resource_acquisition*nutritional_status, data = a)
Anova(AverageMass1)
```

The differences in body size and initial weight of parents among different treatments

```
summary(aov(body_size_male ~ resource_acquisition*nutritional_status, data = a))
summary(aov(body_size_female ~ resource_acquisition*nutritional_status, data = a))
summary(aov(initial_weight_subadult_male ~ resource_acquisition*nutritional_status, data = a))
summary(aov(initial_weight_male ~ resource_acquisition*nutritional_status, data = a))
summary(aov(initial_weight_female ~ resource_acquisition*nutritional_status, data = a))
```
