# Supplementary material for: Access to Abundant Resources Mitigates the Effects of Nutritional Status on Life History Trade‐Offs: An Experimental Study on Burying Beetles
Source: Ecol Evol. 2025 Sep 22;15(9):e72210. doi: 10.1002/ece3.72210 (PMC12453611; doi:10.1002/ece3.72210)
Supplement: Supplementary file 3 — Appendix S3: ece372210‐sup‐0003‐AppendixS3.pdf. [file ECE3-15-e72210-s003.pdf]

Access to abundant resources mitigates the effects of nutritional status on life history trade-offs: an experimental study on burying beetles.

Wenxia Wang, Guojun Zhou, Kai Tian, Lunguang Yao, Jan Komdeur

```
## Packages
library(tidyverse);library(ggpattern);library(readxl);library(ggpmisc);library(ggpubr)
## Data
a <- read_excel("Data.xlsx", col_types = c("text","text","numeric","numeric","numeric",
                                          "numeric","numeric","numeric","numeric","numeric","numeric",
                                          "numeric","numeric","numeric","numeric","numeric","numeric",
                                          "numeric","numeric","numeric","numeric","numeric","numeric"))
```

Figure 1

```
##Figure 1a
p1a<-
  ggplot(a, aes(resource_acquisition, male_care_rate, pattern = resource_acquisition,
                fill = nutritional_status)) +
  stat_boxplot(coef=1000, geom ='errorbar', size = 0.6, width = 0.2,
               position=position_dodge(width = 0.7)) +
  geom_boxplot_pattern(aes(pattern = resource_acquisition),
                       pattern_fill = "black",
                       pattern_angle = 45, pattern_density = 0.1, pattern_spacing = 0.025,
                       pattern_size = 0.1, width = 0.2, size = 0.6, outlier.shape = NA,
                       position=position_dodge(width = 0.7)) +
  scale_fill_manual(name = NULL, labels = c("Poor-fed","Well-fed"), values = c("#EEB4B4", "#6495ED")) +
  scale_pattern_manual(name = NULL, labels = c("8g","15g"), values = c("none", "stripe")) +
  guides(pattern = "none",
         fill = guide_legend(override.aes = list(pattern = "none"))) +
  theme_classic() +
  coord_cartesian(ylim = c(0, 1.2)) +
  scale_y_continuous(breaks = c(0, 0.25, 0.5, 0.75, 1)) +
  scale_x_discrete(labels = c("Small", "Large")) +
  labs(x = "Resource acquisition", y = "Amount of parental care (%)") +
  theme(legend.position = c(0.13, 0.08),
        legend.key.size = unit(16, "pt"),
        legend.text = element_text(size = 10)) +
  theme(axis.text.x = element_text(size = 14)) +
  theme(axis.text.y = element_text(size = 14)) +
  theme(axis.title.x = element_text(size = 14)) +
  theme(axis.title.y = element_text(size = 14)) +
  theme(panel.border = element_rect(color = "black", size = 0.7, fill = NA)) +
  geom_line(data = tibble(x = c(0.83, 1.18), y = c(1, 1)),
```

```r
          aes(x = x, y = y), size = 0.5, inherit.aes = FALSE) +
geom_line(data = tibble(x = c(0.83, 0.83), y = c(0.98, 1)),
          aes(x = x, y = y), size = 0.5, inherit.aes = FALSE) +
geom_line(data = tibble(x = c(1.18, 1.18), y = c(0.98, 1)),
          aes(x = x, y = y), size = 0.5, inherit.aes = FALSE) +
geom_text(data = tibble(x = 1, y = 1.01),
          aes(x = x, y = y, label = "*"), size = 6,
          inherit.aes = FALSE) +
geom_line(data = tibble(x = c(1.83, 2.18), y = c(1, 1)),
          aes(x = x, y = y), size = 0.5, inherit.aes = FALSE) +
geom_line(data = tibble(x = c(1.83, 1.83), y = c(0.98, 1)),
          aes(x = x, y = y), size = 0.5, inherit.aes = FALSE) +
geom_line(data = tibble(x = c(2.18, 2.18), y = c(0.98, 1)),
          aes(x = x, y = y), size = 0.5, inherit.aes = FALSE) +
geom_text(data = tibble(x = 2, y = 1.03),
          aes(x = x, y = y, label = "n.s."), size = 3.8,
          inherit.aes = FALSE) +
geom_line(data = tibble(x = c(0.83, 1.83), y = c(1.05, 1.05)),
          aes(x = x, y = y), size = 0.5, inherit.aes = FALSE) +
geom_line(data = tibble(x = c(0.83, 0.83), y = c(1.03, 1.05)),
          aes(x = x, y = y), size = 0.5, inherit.aes = FALSE) +
geom_line(data = tibble(x = c(1.83, 1.83), y = c(1.03, 1.05)),
          aes(x = x, y = y), size = 0.5, inherit.aes = FALSE) +
geom_text(data = tibble(x = 1.33, y = 1.06),
          aes(x = x, y = y, label = "*"), size = 6,
          inherit.aes = FALSE) +
geom_line(data = tibble(x = c(1.18, 2.18), y = c(1.1, 1.1)),
          aes(x = x, y = y), size = 0.5, inherit.aes = FALSE) +
geom_line(data = tibble(x = c(1.18, 1.18), y = c(1.08, 1.1)),
          aes(x = x, y = y), size = 0.5, inherit.aes = FALSE) +
geom_line(data = tibble(x = c(2.18, 2.18), y = c(1.08, 1.1)),
          aes(x = x, y = y), size = 0.5, inherit.aes = FALSE) +
geom_text(data = tibble(x = 1.68, y = 1.11),
          aes(x = x, y = y, label = "*"), size = 6,
          inherit.aes = FALSE) +
geom_text(data = tibble(x = 0.83, y = 0.7),
          aes(x = x, y = y, label = "25"), size = 3,
          inherit.aes = FALSE) +
geom_text(data = tibble(x = 1.18, y = 0.7),
          aes(x = x, y = y, label = "27"), size = 3,
          inherit.aes = FALSE) +
geom_text(data = tibble(x = 1.83, y = 0.84),
          aes(x = x, y = y, label = "28"), size = 3,
          inherit.aes = FALSE) +
geom_text(data = tibble(x = 2.18, y = 0.83),
          aes(x = x, y = y, label = "27"), size = 3,
            inherit.aes = FALSE) +
geom_point(aes(color = nutritional_status, group = nutritional_status),
           position = position_jitterdodge(jitter.width = 0.1, dodge.width = 0.7),
           size = 1, alpha = 1) +
scale_color_manual(values = c("#FF0000", "#0000FF")) +
guides(color = "none")
```

```r
##Figure 1b
p1b<-
  ggplot(a, aes(resource_acquisition, male_day, pattern = resource_acquisition,
                fill = nutritional_status)) +
  stat_boxplot(coef=1000, geom ='errorbar', size = 0.6, width = 0.2,
               position= position_dodge(width = 0.7)) +
  geom_boxplot_pattern(aes(pattern = resource_acquisition),
                       pattern_fill = "black",
                       pattern_angle = 45, pattern_density = 0.1, pattern_spacing = 0.025,
                       pattern_size = 0.1, width = 0.2, size = 0.6, outlier.shape = NA,
                       position=position_dodge(width = 0.7)) +
  scale_fill_manual(name = NULL, labels = c("Poor-fed","Well-fed"), values = c("#EEB4B4", "#6495ED")) +
  scale_pattern_manual(name = NULL, labels = c("8g","15g"), values = c("none", "stripe")) +
  guides(pattern = "none",
         fill = guide_legend(override.aes = list(pattern = "none"))) +
  theme_classic() +
  coord_cartesian(ylim = c(0, 12)) +
  scale_y_continuous(breaks = c(0, 2.5, 5, 7.5, 10)) +
  scale_x_discrete(labels = c("Small", "Large")) +
  labs(x = "Resource acquisition", y = "Duration of parental care (days)") +
  theme(legend.position = c(0.13, 0.08),
        legend.key.size = unit(16, "pt"),
        legend.text = element_text(size = 10)) +
  theme(axis.text.x = element_text(size = 14)) +
  theme(axis.text.y = element_text(size = 14)) +
  theme(axis.title.x = element_text(size = 14)) +
  theme(axis.title.y = element_text(size = 14)) +
  theme(panel.border = element_rect(color = "black", size = 0.7, fill = NA)) +
  geom_line(data = tibble(x = c(0.83, 1.18), y = c(10.8, 10.8)),
            aes(x = x, y = y), size = 0.5, inherit.aes = FALSE) +
  geom_line(data = tibble(x = c(0.83, 0.83), y = c(10.6, 10.8)),
            aes(x = x, y = y), size = 0.5, inherit.aes = FALSE) +
  geom_line(data = tibble(x = c(1.18, 1.18), y = c(10.6, 10.8)),
            aes(x = x, y = y), size = 0.5, inherit.aes = FALSE) +
  geom_text(data = tibble(x = 1, y = 10.9),
            aes(x = x, y = y, label = "*"), size = 6,
            inherit.aes = FALSE) +
  geom_line(data = tibble(x = c(1.83, 2.18), y = c(10.8, 10.8)),
            aes(x = x, y = y), size = 0.5, inherit.aes = FALSE) +
  geom_line(data = tibble(x = c(1.83, 1.83), y = c(10.6, 10.8)),
            aes(x = x, y = y), size = 0.5, inherit.aes = FALSE) +
  geom_line(data = tibble(x = c(2.18, 2.18), y = c(10.6, 10.8)),
            aes(x = x, y = y), size = 0.5, inherit.aes = FALSE) +
  geom_text(data = tibble(x = 2, y = 10.9),
            aes(x = x, y = y, label = "*"), size = 6,
            inherit.aes = FALSE) +
  geom_line(data = tibble(x = c(0.83, 1.83), y = c(11.3, 11.3)),
            aes(x = x, y = y), size = 0.5, inherit.aes = FALSE) +
  geom_line(data = tibble(x = c(0.83, 0.83), y = c(11.1, 11.3)),
            aes(x = x, y = y), size = 0.5, inherit.aes = FALSE) +
  geom_line(data = tibble(x = c(1.83, 1.83), y = c(11.1, 11.3)),
            aes(x = x, y = y), size = 0.5, inherit.aes = FALSE) +
  geom_text(data = tibble(x = 1.33, y = 11.4),
```

```
           aes(x = x, y = y, label = "*"), size = 6,
           inherit.aes = FALSE) +
  geom_line(data = tibble(x = c(1.18, 2.18), y = c(11.8, 11.8)),
           aes(x = x, y = y), size = 0.5, inherit.aes = FALSE) +
  geom_line(data = tibble(x = c(1.18, 1.18), y = c(11.6, 11.8)),
           aes(x = x, y = y), size = 0.5, inherit.aes = FALSE) +
  geom_line(data = tibble(x = c(2.18, 2.18), y = c(11.6, 11.8)),
           aes(x = x, y = y), size = 0.5, inherit.aes = FALSE) +
  geom_text(data = tibble(x = 1.68, y = 11.9),
           aes(x = x, y = y, label = "*"), size = 6,
           inherit.aes = FALSE) +
  geom_text(data = tibble(x = 0.83, y = 9.4),
           aes(x = x, y = y, label = "25"), size = 3,
           inherit.aes = FALSE) +
  geom_text(data = tibble(x = 1.18, y = 8.4),
           aes(x = x, y = y, label = "27"), size = 3,
           inherit.aes = FALSE) +
  geom_text(data = tibble(x = 1.83, y = 10.3),
           aes(x = x, y = y, label = "28"), size = 3,
           inherit.aes = FALSE) +
  geom_text(data = tibble(x = 2.18, y = 10.3),
           aes(x = x, y = y, label = "27"), size = 3,
           inherit.aes = FALSE) +
  geom_point(aes(color = nutritional_status, group = nutritional_status),
            position = position_jitterdodge(jitter.width = 0.1, dodge.width = 0.7),
            size = 1, alpha = 1) +
  scale_color_manual(values = c("#FF0000", "#0000FF")) +
  guides(color = "none")


##Figure 1c
p1c<-
  ggplot(a, aes(resource_acquisition, final_weight_male, pattern = resource_acquisition,
              fill = nutritional_status)) +
  stat_boxplot(coef=1000, geom ='errorbar', size = 0.6, width = 0.2,
             position= position_dodge(width = 0.7)) +
  geom_boxplot_pattern(aes(pattern = resource_acquisition),
                     pattern_fill = "black",
                     pattern_angle = 45, pattern_density = 0.1, pattern_spacing = 0.025,
                     pattern_size = 0.1, width = 0.2, size = 0.6, outlier.shape = NA,
                     position=position_dodge(width = 0.7)) +
  scale_fill_manual(name = NULL, labels = c("Poor-fed","Well-fed"), values = c("#EEB4B4", "#6495ED")) +
  scale_pattern_manual(name = NULL, labels = c("8g","15g"), values = c("none", "stripe")) +
  guides(pattern = "none",
        fill = guide_legend(override.aes = list(pattern = "none"), order = 0)) +
  theme_classic() +
  coord_cartesian(ylim = c(0, 0.25)) +
  scale_y_continuous(breaks = c(0, 0.05, 0.10, 0.15, 0.20)) +
  scale_x_discrete(labels = c("Small", "Large")) +
  labs(x = "Resource acquisition", y = "Final weight of males (g)") +
  theme(legend.position = c(0.13, 0.08),
      legend.key.size = unit(16, "pt"),
      legend.text = element_text(size = 10)) +
```

```r
theme(axis.text.x = element_text(size = 14)) +
theme(axis.text.y = element_text(size = 14)) +
theme(axis.title.x = element_text(size = 14)) +
theme(axis.title.y = element_text(size = 14)) +
theme(panel.border = element_rect(color = "black", size = 0.7, fill = NA)) +
geom_line(data = tibble(x = c(0.83, 1.18), y = c(0.225, 0.225)),
          aes(x = x, y = y), size = 0.5, inherit.aes = FALSE) +
geom_line(data = tibble(x = c(0.83, 0.83), y = c(0.22, 0.225)),
          aes(x = x, y = y), size = 0.5, inherit.aes = FALSE) +
geom_line(data = tibble(x = c(1.18, 1.18), y = c(0.22, 0.225)),
          aes(x = x, y = y), size = 0.5, inherit.aes = FALSE) +
geom_text(data = tibble(x = 1, y = 0.227),
          aes(x = x, y = y, label = "*"), size = 6,
          inherit.aes = FALSE) +
geom_line(data = tibble(x = c(1.83, 2.18), y = c(0.225, 0.225)),
          aes(x = x, y = y), size = 0.5, inherit.aes = FALSE) +
geom_line(data = tibble(x = c(1.83, 1.83), y = c(0.22, 0.225)),
          aes(x = x, y = y), size = 0.5, inherit.aes = FALSE) +
geom_line(data = tibble(x = c(2.18, 2.18), y = c(0.22, 0.225)),
          aes(x = x, y = y), size = 0.5, inherit.aes = FALSE) +
geom_text(data = tibble(x = 2, y = 0.233),
          aes(x = x, y = y, label = "n.s."), size = 3.8,
          inherit.aes = FALSE) +
geom_line(data = tibble(x = c(0.83, 1.83), y = c(0.236, 0.236)),
          aes(x = x, y = y), size = 0.5, inherit.aes = FALSE) +
geom_line(data = tibble(x = c(0.83, 0.83), y = c(0.231, 0.236)),
          aes(x = x, y = y), size = 0.5, inherit.aes = FALSE) +
geom_line(data = tibble(x = c(1.83, 1.83), y = c(0.231, 0.236)),
          aes(x = x, y = y), size = 0.5, inherit.aes = FALSE) +
geom_text(data = tibble(x = 1.33, y = 0.238),
          aes(x = x, y = y, label = "*"), size = 6,
          inherit.aes = FALSE) +
geom_line(data = tibble(x = c(1.18, 2.18), y = c(0.246, 0.246)),
          aes(x = x, y = y), size = 0.5, inherit.aes = FALSE) +
geom_line(data = tibble(x = c(1.18, 1.18), y = c(0.241, 0.246)),
          aes(x = x, y = y), size = 0.5, inherit.aes = FALSE) +
geom_line(data = tibble(x = c(2.18, 2.18), y = c(0.241, 0.246)),
          aes(x = x, y = y), size = 0.5, inherit.aes = FALSE) +
geom_text(data = tibble(x = 1.68, y = 0.248),
          aes(x = x, y = y, label = "*"), size = 6,
          inherit.aes = FALSE) +
geom_text(data = tibble(x = 0.83, y = 0.175),
          aes(x = x, y = y, label = "25"), size = 3,
          inherit.aes = FALSE) +
geom_text(data = tibble(x = 1.18, y = 0.205),
          aes(x = x, y = y, label = "27"), size = 3,
          inherit.aes = FALSE) +
geom_text(data = tibble(x = 1.83, y = 0.216),
          aes(x = x, y = y, label = "28"), size = 3,
          inherit.aes = FALSE) +
geom_text(data = tibble(x = 2.18, y = 0.204),
          aes(x = x, y = y, label = "27"), size = 3,
          inherit.aes = FALSE) +
```

```
  geom_point(aes(color = nutritional_status, group = nutritional_status),
             position = position_jitterdodge(jitter.width = 0.1, dodge.width = 0.7),
             size = 1, alpha = 1) +
  scale_color_manual(values = c("#FF0000", "#0000FF")) +
  guides(color = "none")


##Figure 1d
p1d<-
  ggplot(a, aes(resource_acquisition, weight_change_male, pattern = resource_acquisition,
                fill = nutritional_status)) +
  stat_boxplot(coef=1000, geom ='errorbar', size = 0.6, width = 0.2,
                position= position_dodge(width = 0.7)) +
  geom_boxplot_pattern(aes(pattern = resource_acquisition),
                pattern_fill = "black",
                pattern_angle = 45, pattern_density = 0.1, pattern_spacing = 0.025,
                pattern_size = 0.1, width = 0.2, size = 0.6, outlier.shape = NA,
                position=position_dodge(width = 0.7)) +
  scale_fill_manual(name = NULL, labels = c("Poor-fed","Well-fed"), values = c("#EEB4B4", "#6495ED")) +
  scale_pattern_manual(name = NULL, labels = c("8g","15g"), values = c("none", "stripe")) +
  guides(pattern = "none",
                fill = guide_legend(override.aes = list(pattern = "none"), order = 0)) +
  theme_classic() +
  coord_cartesian(ylim = c(0, 0.07)) +
  scale_y_continuous(breaks = c(0, 0.015, 0.03, 0.045, 0.06)) +
  scale_x_discrete(labels = c("Small", "Large")) +
  labs(x = "Resource acquisition", y = "Weight change of males (g)") +
  theme(legend.position = c(0.13, 0.08),
        legend.key.size = unit(16, "pt"),
        legend.text = element_text(size = 10)) +
  theme(axis.text.x = element_text(size = 14)) +
  theme(axis.text.y = element_text(size = 14)) +
  theme(axis.title.x = element_text(size = 14)) +
  theme(axis.title.y = element_text(size = 14)) +
  theme(panel.border = element_rect(color = "black", size = 0.7, fill = NA)) +
  geom_line(data = tibble(x = c(0.83, 1.18), y = c(0.06, 0.06)),
            aes(x = x, y = y), size = 0.5, inherit.aes = FALSE) +
  geom_line(data = tibble(x = c(0.83, 0.83), y = c(0.058, 0.06)),
            aes(x = x, y = y), size = 0.5, inherit.aes = FALSE) +
  geom_line(data = tibble(x = c(1.18, 1.18), y = c(0.058, 0.06)),
            aes(x = x, y = y), size = 0.5, inherit.aes = FALSE) +
  geom_text(data = tibble(x = 1, y = 0.0605),
            aes(x = x, y = y, label = "*"), size = 6,
            inherit.aes = FALSE) +
  geom_line(data = tibble(x = c(1.83, 2.18), y = c(0.06, 0.06)),
            aes(x = x, y = y), size = 0.5, inherit.aes = FALSE) +
  geom_line(data = tibble(x = c(1.83, 1.83), y = c(0.058, 0.06)),
            aes(x = x, y = y), size = 0.5, inherit.aes = FALSE) +
  geom_line(data = tibble(x = c(2.18, 2.18), y = c(0.058, 0.06)),
            aes(x = x, y = y), size = 0.5, inherit.aes = FALSE) +
  geom_text(data = tibble(x = 2, y = 0.0605),
            aes(x = x, y = y, label = "*"), size = 6,
            inherit.aes = FALSE) +
```

```
  geom_line(data = tibble(x = c(0.83, 1.83), y = c(0.063, 0.063)),
            aes(x = x, y = y), size = 0.5, inherit.aes = FALSE) +
  geom_line(data = tibble(x = c(0.83, 0.83), y = c(0.061, 0.063)),
            aes(x = x, y = y), size = 0.5, inherit.aes = FALSE) +
  geom_line(data = tibble(x = c(1.83, 1.83), y = c(0.061, 0.063)),
            aes(x = x, y = y), size = 0.5, inherit.aes = FALSE) +
  geom_text(data = tibble(x = 1.33, y = 0.0635),
            aes(x = x, y = y, label = "*"), size = 6,
            inherit.aes = FALSE) +
  geom_line(data = tibble(x = c(1.18, 2.18), y = c(0.066, 0.066)),
            aes(x = x, y = y), size = 0.5, inherit.aes = FALSE) +
  geom_line(data = tibble(x = c(1.18, 1.18), y = c(0.064, 0.066)),
            aes(x = x, y = y), size = 0.5, inherit.aes = FALSE) +
  geom_line(data = tibble(x = c(2.18, 2.18), y = c(0.064, 0.066)),
            aes(x = x, y = y), size = 0.5, inherit.aes = FALSE) +
  geom_text(data = tibble(x = 1.68, y = 0.068),
            aes(x = x, y = y, label = "n.s."), size = 3.8,
            inherit.aes = FALSE) +
  geom_text(data = tibble(x = 0.83, y = 0.0347),
            aes(x = x, y = y, label = "25"), size = 3,
            inherit.aes = FALSE) +
  geom_text(data = tibble(x = 1.18, y = 0.0360),
            aes(x = x, y = y, label = "27"), size = 3,
            inherit.aes = FALSE) +
  geom_text(data = tibble(x = 1.83, y = 0.0559),
            aes(x = x, y = y, label = "28"), size = 3,
            inherit.aes = FALSE) +
  geom_text(data = tibble(x = 2.18, y = 0.0335),
            aes(x = x, y = y, label = "27"), size = 3,
            inherit.aes = FALSE) +
  geom_point(aes(color = nutritional_status, group = nutritional_status),
             position = position_jitterdodge(jitter.width = 0.1, dodge.width = 0.7),
             size = 1, alpha = 1) +
  scale_color_manual(values = c("#FF0000", "#0000FF")) +
  guides(color = "none")

##Save Figure 1
  ggarrange(p1a, p1b, p1c, p1d, ncol = 2, nrow = 2,
            labels = c("a","b","c","d"),
            label.x = 0.01, label.y = 0.97,
            font.label = list(size = 16, face = "bold"))
```

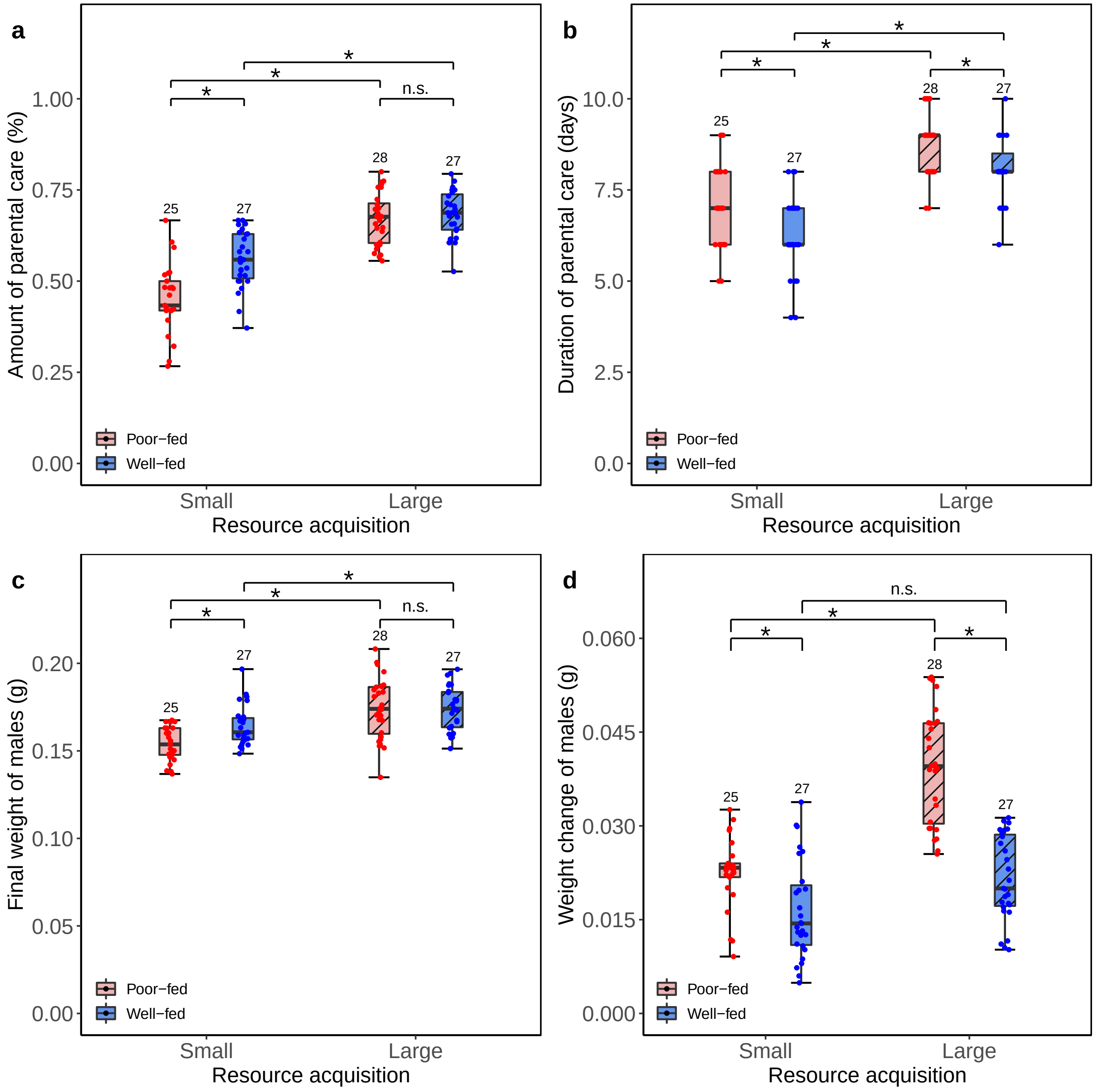


```
ggsave("FIGURE 1.tiff", width = 10, height = 10)
```

Figure 2

```
##Figure 2a
p2a<-
  ggplot(a, aes(resource_acquisition, larvae_number, pattern = resource_acquisition,
                fill = nutritional_status)) +
  stat_boxplot(coef=1000, geom ='errorbar', size = 0.6, width = 0.2,
               position= position_dodge(width = 0.7)) +
  geom_boxplot_pattern(aes(pattern = resource_acquisition),
                       pattern_fill = "black",
```

```
                       pattern_angle = 45, pattern_density = 0.1, pattern_spacing = 0.025,
                       pattern_size = 0.1, width = 0.2, size = 0.6, outlier.shape = NA,
                       position=position_dodge(width = 0.7)) +
scale_fill_manual(name = NULL, labels = c("Poor-fed","Well-fed"), values = c("#EEB4B4", "#6495ED")) +
scale_pattern_manual(name = NULL, labels = c("8g","15g"), values = c("none", "stripe")) +
guides(pattern = "none",
       fill = guide_legend(override.aes = list(pattern = "none"), order = 0)) +
theme_classic() +
coord_cartesian(ylim = c(0, 28)) +
scale_y_continuous(breaks = c(0, 5, 10, 15, 20, 25)) +
scale_x_discrete(labels = c("Small", "Large")) +
labs(x = "Resource acquisition", y = "Larvae number") +
theme(legend.position = c(0.13, 0.08),
      legend.key.size = unit(16, "pt"),
      legend.text = element_text(size = 10)) +
theme(axis.text.x = element_text(size = 14)) +
theme(axis.text.y = element_text(size = 14)) +
theme(axis.title.x = element_text(size = 14)) +
theme(axis.title.y = element_text(size = 14)) +
theme(legend.text = element_text(size = 10)) +
theme(legend.key.size = unit(13, "pt")) +
theme(panel.border = element_rect(color = "black", size = 0.7, fill = NA)) +
geom_line(data = tibble(x = c(0.83, 1.18), y = c(25, 25)),
          aes(x = x, y = y), size = 0.5, inherit.aes = FALSE) +
geom_line(data = tibble(x = c(0.83, 0.83), y = c(24.3, 25)),
          aes(x = x, y = y), size = 0.5, inherit.aes = FALSE) +
geom_line(data = tibble(x = c(1.18, 1.18), y = c(24.3, 25)),
          aes(x = x, y = y), size = 0.5, inherit.aes = FALSE) +
geom_text(data = tibble(x = 1, y = 25.9),
          aes(x = x, y = y, label = "n.s."), size = 3.8,
          inherit.aes = FALSE) +
geom_line(data = tibble(x = c(1.83, 2.18), y = c(25, 25)),
          aes(x = x, y = y), size = 0.5, inherit.aes = FALSE) +
geom_line(data = tibble(x = c(1.83, 1.83), y = c(24.3, 25)),
          aes(x = x, y = y), size = 0.5, inherit.aes = FALSE) +
geom_line(data = tibble(x = c(2.18, 2.18), y = c(24.3, 25)),
          aes(x = x, y = y), size = 0.5, inherit.aes = FALSE) +
geom_text(data = tibble(x = 2, y = 25.9),
          aes(x = x, y = y, label = "n.s."), size = 3.8,
          inherit.aes = FALSE) +
geom_line(data = tibble(x = c(0.83, 1.83), y = c(26.5, 26.5)),
          aes(x = x, y = y), size = 0.5, inherit.aes = FALSE) +
geom_line(data = tibble(x = c(0.83, 0.83), y = c(25.8, 26.5)),
          aes(x = x, y = y), size = 0.5, inherit.aes = FALSE) +
geom_line(data = tibble(x = c(1.83, 1.83), y = c(25.8, 26.5)),
          aes(x = x, y = y), size = 0.5, inherit.aes = FALSE) +
geom_text(data = tibble(x = 1.33, y = 26.7),
          aes(x = x, y = y, label = "*"), size = 6,
          inherit.aes = FALSE) +
geom_line(data = tibble(x = c(1.18, 2.18), y = c(27.7, 27.7)),
          aes(x = x, y = y), size = 0.5, inherit.aes = FALSE) +
geom_line(data = tibble(x = c(1.18, 1.18), y = c(27, 27.7)),
          aes(x = x, y = y), size = 0.5, inherit.aes = FALSE) +
```

```r
  geom_line(data = tibble(x = c(2.18, 2.18), y = c(27, 27.7)),
            aes(x = x, y = y), size = 0.5, inherit.aes = FALSE) +
  geom_text(data = tibble(x = 1.68, y = 27.9),
            aes(x = x, y = y, label = "*"), size = 6,
            inherit.aes = FALSE) +
  geom_text(data = tibble(x = 0.83, y = 16.8),
            aes(x = x, y = y, label = "25"), size = 3,
            inherit.aes = FALSE) +
  geom_text(data = tibble(x = 1.18, y = 16.8),
            aes(x = x, y = y, label = "27"), size = 3,
            inherit.aes = FALSE) +
  geom_text(data = tibble(x = 1.83, y = 23.8),
            aes(x = x, y = y, label = "28"), size = 3,
            inherit.aes = FALSE) +
  geom_text(data = tibble(x = 2.18, y = 21.8),
            aes(x = x, y = y, label = "27"), size = 3,
            inherit.aes = FALSE) +
  geom_point(aes(color = nutritional_status, group = nutritional_status),
             position = position_jitterdodge(jitter.width = 0.1, dodge.width = 0.7),
             size = 1, alpha = 1) +
  scale_color_manual(values = c("#FF0000", "#0000FF")) +
  guides(color = "none")


##Figure 2b
p2b<-
  ggplot(a, aes(resource_acquisition, average_larvae_mass, pattern = resource_acquisition,
              fill = nutritional_status))+
  stat_boxplot(coef=1000, geom ='errorbar', size = 0.6, width = 0.2,
             position= position_dodge(width = 0.7)) +
  geom_boxplot_pattern(aes(pattern = resource_acquisition),
                     pattern_fill = "black",
                     pattern_angle = 45, pattern_density = 0.1, pattern_spacing = 0.025,
                     pattern_size = 0.1, width = 0.2, size = 0.6, outlier.shape = NA,
                     position=position_dodge(width = 0.7)) +
  scale_fill_manual(name = NULL, labels = c("Poor-fed","Well-fed"), values = c("#EEB4B4", "#6495ED")) +
  scale_pattern_manual(name = NULL, labels = c("8g","15g"), values = c("none", "stripe")) +
  guides(pattern = "none",
       fill = guide_legend(override.aes = list(pattern = "none"), order = 0)) +
  theme_classic() +
  coord_cartesian(ylim = c(0, 0.23)) +
  scale_y_continuous(breaks = c(0, 0.05, 0.1, 0.15, 0.2)) +
  scale_x_discrete(labels = c("Small", "Large")) +
  labs(x = "Resource acquisition", y = "Average larval mass (g)") +
  theme(legend.position = c(0.13, 0.08),
      legend.key.size = unit(16, "pt"),
      legend.text = element_text(size = 10)) +
  theme(axis.text.x = element_text(size = 14)) +
  theme(axis.text.y = element_text(size = 14)) +
  theme(axis.title.x = element_text(size = 14)) +
  theme(axis.title.y = element_text(size = 14)) +
  theme(legend.text = element_text(size = 10)) +
  theme(legend.key.size = unit(13, "pt")) +
```

```r
theme(panel.border = element_rect(color = "black", size = 0.7, fill = NA)) +
geom_line(data = tibble(x = c(0.83, 1.18), y = c(0.205, 0.205)),
          aes(x = x, y = y), size = 0.5, inherit.aes = FALSE) +
geom_line(data = tibble(x = c(0.83, 0.83), y = c(0.2, 0.205)),
          aes(x = x, y = y), size = 0.5, inherit.aes = FALSE) +
geom_line(data = tibble(x = c(1.18, 1.18), y = c(0.2, 0.205)),
          aes(x = x, y = y), size = 0.5, inherit.aes = FALSE) +
geom_text(data = tibble(x = 1, y = 0.212),
          aes(x = x, y = y, label = "n.s."), size = 3.8,
          inherit.aes = FALSE) +
geom_line(data = tibble(x = c(1.83, 2.18), y = c(0.205, 0.205)),
          aes(x = x, y = y), size = 0.5, inherit.aes = FALSE) +
geom_line(data = tibble(x = c(1.83, 1.83), y = c(0.2, 0.205)),
          aes(x = x, y = y), size = 0.5, inherit.aes = FALSE) +
geom_line(data = tibble(x = c(2.18, 2.18), y = c(0.2, 0.205)),
          aes(x = x, y = y), size = 0.5, inherit.aes = FALSE) +
geom_text(data = tibble(x = 2, y = 0.212),
          aes(x = x, y = y, label = "n.s."), size = 3.8,
          inherit.aes = FALSE) +
geom_line(data = tibble(x = c(0.83, 1.83), y = c(0.217, 0.217)),
          aes(x = x, y = y), size = 0.5, inherit.aes = FALSE) +
geom_line(data = tibble(x = c(0.83, 0.83), y = c(0.212, 0.217)),
          aes(x = x, y = y), size = 0.5, inherit.aes = FALSE) +
geom_line(data = tibble(x = c(1.83, 1.83), y = c(0.212, 0.217)),
          aes(x = x, y = y), size = 0.5, inherit.aes = FALSE) +
geom_text(data = tibble(x = 1.33, y = 0.219),
          aes(x = x, y = y, label = "*"), size = 6,
          inherit.aes = FALSE) +
geom_line(data = tibble(x = c(1.18, 2.18), y = c(0.227, 0.227)),
          aes(x = x, y = y), size = 0.5, inherit.aes = FALSE) +
geom_line(data = tibble(x = c(1.18, 1.18), y = c(0.222, 0.227)),
          aes(x = x, y = y), size = 0.5, inherit.aes = FALSE) +
geom_line(data = tibble(x = c(2.18, 2.18), y = c(0.222, 0.227)),
          aes(x = x, y = y), size = 0.5, inherit.aes = FALSE) +
geom_text(data = tibble(x = 1.68, y = 0.229),
          aes(x = x, y = y, label = "*"), size = 6,
          inherit.aes = FALSE) +

geom_text(data = tibble(x = 0.83, y = 0.17),
          aes(x = x, y = y, label = "25"), size = 3,
          inherit.aes = FALSE) +
geom_text(data = tibble(x = 1.18, y = 0.155),
          aes(x = x, y = y, label = "27"), size = 3,
          inherit.aes = FALSE) +
geom_text(data = tibble(x = 1.83, y = 0.196),
          aes(x = x, y = y, label = "28"), size = 3,
          inherit.aes = FALSE) +
geom_text(data = tibble(x = 2.18, y = 0.194),
          aes(x = x, y = y, label = "27"), size = 3,
          inherit.aes = FALSE) +
geom_point(aes(color = nutritional_status, group = nutritional_status),
          position = position_jitterdodge(jitter.width = 0.1, dodge.width = 0.7),
          size = 1, alpha = 1) +
```

```
  scale_color_manual(values = c("#FF0000", "#0000FF")) +
  guides(color = "none")


##Figure 2c the default fits produced by ggplot
p2c<-
ggplot(a, aes(larvae_number, average_larvae_mass, color = interaction, linetype = interaction)) +
  geom_point(size = 1, shape = 16)+
  geom_smooth(method = "lm", se = T, size = 0.6) +
  scale_color_manual(values = c("#000000", "#0000FF", "#FF0000", "#696969"),
                     labels = c("Poor-fed*Small carcass", "Poor-fed*Large carcass",
                                "Well-fed*Small carcass", "Well-fed*Large carcass")) +
  scale_linetype_manual(values = c("solid", "solid", "solid", "dashed"),
                        labels = c("Poor-fed*Small carcass", "Poor-fed*Large carcass",
                                   "Well-fed*Small carcass", "Well-fed*Large carcass")) +
  scale_x_continuous(breaks = c(5, 10, 15, 20, 25)) +
  scale_y_continuous(breaks = c(0.1, 0.15, 0.2)) +
  labs(x = "Larvae number",
       y = "Average larval mass (g)") +
  coord_cartesian(xlim = c(3, 25), ylim = c(0.08, 0.2)) +
  theme_classic() +
  theme(legend.position = c(0.22, 0.13)) +
  theme(legend.title = element_blank()) +
  theme(legend.text = element_text(size = 9)) +
  theme(legend.key.size = unit(16, "pt")) +
  theme(axis.text.x = element_text(size = 14)) +
  theme(axis.text.y = element_text(size = 14)) +
  theme(axis.title.x = element_text(size = 16)) +
  theme(axis.title.y = element_text(size = 16)) +
  theme(panel.border = element_rect(color = "black", size = 0.7, fill = NA)) +
  theme(plot.title = element_text(hjust = -0.17, vjust = -9, size = 13))
# + stat_poly_eq(aes(label =  paste(stat(eq.label),
#                                   stat(adj.rr.label),
#                                   stat(p.value.label),
#                                   sep = "*\", \"*")),
#              label.x = c(0.25, 0.25, 0.25, 0.25), label.y = c(0.15,0.1, 0.05, 0.01),
#              formula = y ~ x, parse = TRUE, size = 3,
#              rr.digits = 5, coef.digits = 5, p.digits = 5) use this code to obtain P values

##Save Figure 2
  ggarrange(p2a, p2b, p2c, ncol = 3, nrow = 1,
            labels = c("a","b","c"),
            label.x = 0.01, label.y = 0.97,
            font.label = list(size = 16, face = "bold"))
```

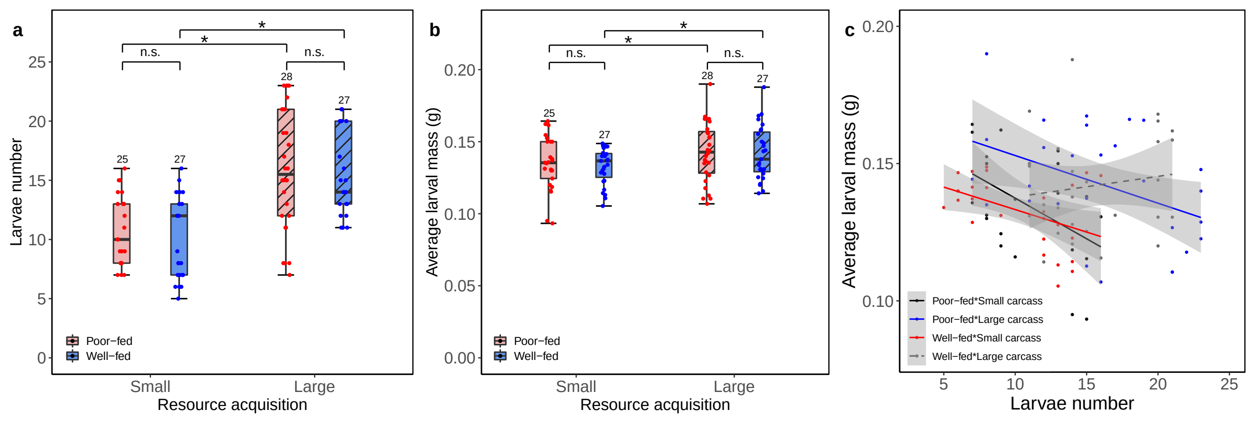


```r
ggsave("FIGURE 2.tiff", width = 15, height = 5)
```

Figure 2c_1, the fits are predictions from the models

```r
library(dplyr);library(tidyr)
 predictions <- a %>%
   group_by(interaction) %>%
   do({
     model <- lm(average_larvae_mass ~ larvae_number, data = .)
     group_data <- data.frame(
       larvae_number = seq(3, 25, length.out = 100))

     pred <- predict(model, newdata = group_data, se.fit = TRUE)
     group_data$predicted_mass <- pred$fit
     group_data$se <- pred$se.fit
     group_data$lower <- pred$fit - 1.96 * pred$se.fit
     group_data$upper <- pred$fit + 1.96 * pred$se.fit
     group_data$interaction <- first(.$interaction)
     group_data
   }) %>%
   ungroup()
 #Use model predicted values to plot the graph
 ggplot() +
   geom_ribbon(data = predictions, aes(x = larvae_number, ymin = lower, ymax = upper,
                                       group = interaction),
               fill = "grey80", alpha = 0.4, show.legend = FALSE) +
   geom_point(data = a, aes(x = larvae_number, y = average_larvae_mass,
                            color = interaction),
              size = 1, shape = 16) +
   geom_line(data = predictions, aes(x = larvae_number, y = predicted_mass,
                                     color = interaction,
                                     linetype = interaction), size = 0.6) +
   scale_color_manual(values = c("#000000", "#0000FF", "#FF0000", "#696969"),
                      labels = c("Poor-fed*Small carcass", "Poor-fed*Large carcass",
                                 "Well-fed*Small carcass", "Well-fed*Large carcass")) +
   scale_linetype_manual(values = c("solid", "solid", "solid", "dashed"),
                         labels = c("Poor-fed*Small carcass", "Poor-fed*Large carcass",
                                    "Well-fed*Small carcass", "Well-fed*Large carcass")) +
```

```r
  scale_x_continuous(breaks = c(5, 10, 15, 20, 25)) +
  scale_y_continuous(breaks = c(0.1, 0.15, 0.2)) +
  labs(x = "Larvae number",
       y = "Average larval mass (g)") +
  coord_cartesian(xlim = c(3, 25), ylim = c(0.08, 0.2)) +
  theme_classic() +
  theme(legend.position = c(0.22, 0.13)) +
  theme(legend.title = element_blank()) +
  theme(legend.text = element_text(size = 9)) +
  theme(legend.key.size = unit(16, "pt")) +
  theme(axis.text.x = element_text(size = 14)) +
  theme(axis.text.y = element_text(size = 14)) +
  theme(axis.title.x = element_text(size = 16)) +
  theme(axis.title.y = element_text(size = 16)) +
  theme(panel.border = element_rect(color = "black", size = 0.7, fill = NA)) +
  theme(plot.title = element_text(hjust = -0.17, vjust = -9, size = 13))
```

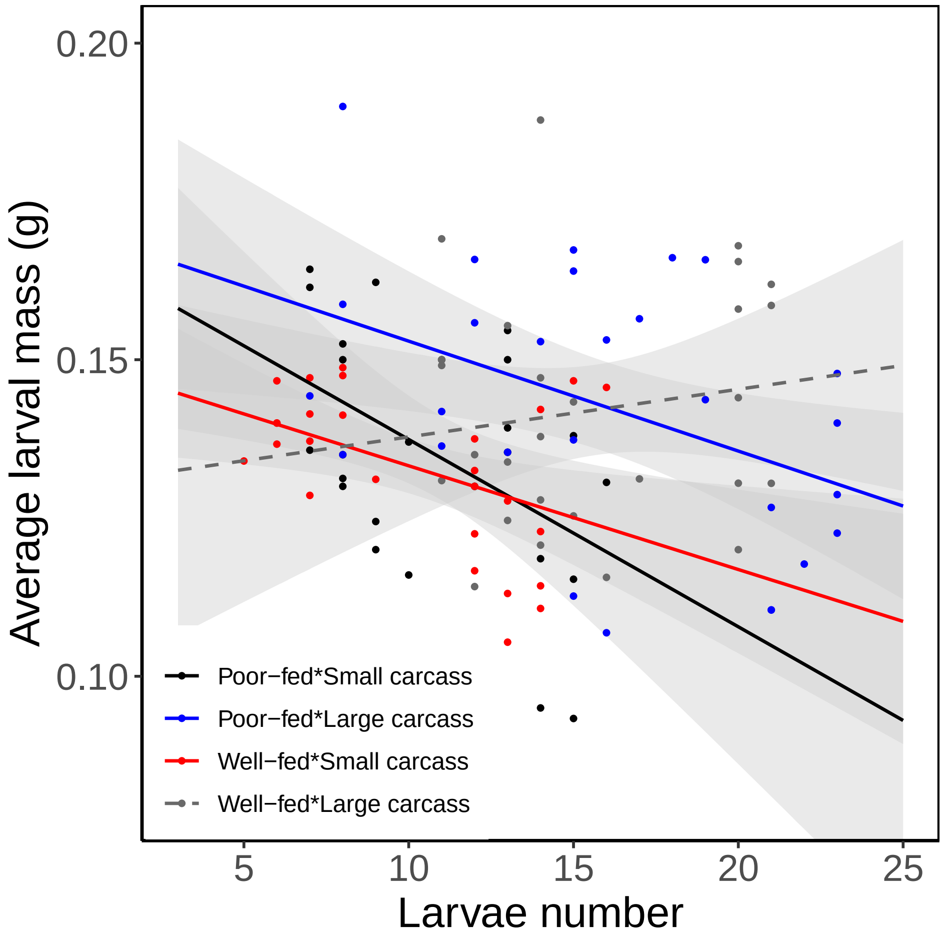


```r
# Extract model statistics for each group
model_stats <- a %>%
  group_by(interaction) %>%
  do({
    model <- lm(average_larvae_mass ~ larvae_number, data = .)
```

```r
    model_summary <- summary(model)
    data.frame(
      interaction = first(.$interaction),
      intercept = coef(model)[1],
      slope = coef(model)[2],
      r_squared = model_summary$r.squared,
      p_value = coef(model_summary)[2, 4]
    )
  }) %>%
  ungroup()          #The P values obtained from these two methods
print(model_stats)   #(model predictions vs default ggplot) are consistent.
```

```
## # A tibble: 4 x 5
##   interaction intercept     slope r_squared p_value
##   <fct>           <dbl>     <dbl>     <dbl>   <dbl>
## 1 8:P             0.167 -0.00296     0.222   0.0175
## 2 15:P            0.170 -0.00174     0.181   0.0241
## 3 8:W             0.150 -0.00164     0.202   0.0188
## 4 15:W            0.130  0.000753    0.0217  0.464
```
