# Supplementary material for: Access to Abundant Resources Mitigates the Effects of Nutritional Status on Life History Trade‐Offs: An Experimental Study on Burying Beetles
Source: Ecol Evol. 2025 Sep 22;15(9):e72210. doi: 10.1002/ece3.72210 (PMC12453611; doi:10.1002/ece3.72210)
Supplement: Supplementary file 4 — Table S1: Descriptive statistics for initial weight and pronotum width of burying beetle parents. Table S2: Descriptive statistics for the amount and duration of parental care, final weight and weight change of burying beetle males, larvae number, and average larval mass at dispersal. [file ECE3-15-e72210-s004.docx]

Supplementary Material

**Table S1.** Descriptive statistics for initial weight and pronotum width of burying beetle parents.

| Explanatory variable | |  | Initial subadult male weight (g) | | Initial adult male weight (g) | | Initial adult female weight (g) | | Male pronotum width (mm) | | Female pronotum width (mm) | |
| --- | --- | --- | --- | --- | --- | --- | --- | --- | --- | --- | --- | --- |
|  |  | *N* | Mean | SD | Mean | SD | Mean | SD | Mean | SD | Mean | SD |
| Nutritional status | Poor-fed | 53 | 0.127 | 0.016 | 0.133 | 0.013 | 0.127 | 0.019 | 4.510 | 0.583 | 4.594 | 0.535 |
|  | Well-fed | 54 | 0.125 | 0.019 | 0.150 | 0.011 | 0.129 | 0.021 | 4.639 | 0.676 | 4.718 | 0.671 |
| Resource acquisition | Small | 52 | 0.124 | 0.017 | 0.139 | 0.015 | 0.129 | 0.023 | 4.568 | 0.658 | 4.626 | 0.600 |
|  | Large | 55 | 0.127 | 0.018 | 0.143 | 0.015 | 0.127 | 0.018 | 4.582 | 0.613 | 4.685 | 0.619 |
| Interaction | Poor-fed*Large | 28 | 0.128 | 0.016 | 0.135 | 0.014 | 0.125 | 0.019 | 4.534 | 0.501 | 4.661 | 0.536 |
|  | Well-fed*Large | 27 | 0.126 | 0.021 | 0.153 | 0.009 | 0.129 | 0.017 | 4.631 | 0.717 | 4.710 | 0.705 |
|  | Poor-fed*Small | 25 | 0.126 | 0.015 | 0.131 | 0.012 | 0.128 | 0.020 | 4.483 | 0.672 | 4.518 | 0.535 |
|  | Well-fed*Small | 27 | 0.122 | 0.018 | 0.147 | 0.012 | 0.129 | 0.025 | 4.646 | 0.647 | 4.727 | 0.648 |

SD, standard deviation; *N*, sample size.

**Table S2.** Descriptive statistics for the amount and duration of parental care, final weight and weight change of burying beetle males, larvae number, and average larval mass at dispersal.

| Explanatory variable | |  | Amount of  parental care (%) | | Duration of  parental care (day) | | Final weight (g) | | Weight change (g) | | Larvae number | | Average larval mass (g) | |
| --- | --- | --- | --- | --- | --- | --- | --- | --- | --- | --- | --- | --- | --- | --- |
|  |  | *N* | Mean | SD | Mean | SD | Mean | SD | Mean | SD | Mean | SD | Mean | SD |
| Nutritional status | Poor-fed | 53 | 0.565 | 0.137 | 7.943 | 1.322 | 0.165 | 0.017 | 0.032 | 0.012 | 13.547 | 4.909 | 0.139 | 0.020 |
|  | Well-fed | 54 | 0.621 | 0.094 | 7.093 | 1.349 | 0.169 | 0.013 | 0.019 | 0.008 | 12.944 | 4.470 | 0.137 | 0.016 |
| Resource acquisition | Small | 52 | 0.505 | 0.099 | 6.558 | 1.162 | 0.159 | 0.012 | 0.019 | 0.007 | 10.539 | 3.220 | 0.134 | 0.016 |
|  | Large | 55 | 0.677 | 0.067 | 8.418 | 0.917 | 0.174 | 0.015 | 0.031 | 0.012 | 15.800 | 4.416 | 0.142 | 0.020 |
| Interaction | Poor-fed*Large | 28 | 0.670 | 0.071 | 8.785 | 0.833 | 0.174 | 0.017 | 0.040 | 0.009 | 15.929 | 5.098 | 0.143 | 0.021 |
|  | Well-fed*Large | 27 | 0.684 | 0.063 | 8.037 | 0.854 | 0.174 | 0.013 | 0.022 | 0.007 | 15.667 | 3.669 | 0.142 | 0.019 |
|  | Poor-fed*Small | 25 | 0.448 | 0.097 | 7.000 | 1.118 | 0.154 | 0.009 | 0.022 | 0.006 | 10.880 | 2.991 | 0.135 | 0.019 |
|  | Well-fed*Small | 27 | 0.559 | 0.078 | 6.148 | 1.064 | 0.164 | 0.011 | 0.016 | 0.008 | 10.222 | 3.446 | 0.133 | 0.013 |

SD, standard deviation; *N*, sample size.
